# Abnormal Expression and Prognostic Significance of Bone Morphogenetic Proteins and Their Receptors in Lung Adenocarcinoma

**DOI:** 10.1155/2021/6663990

**Published:** 2021-05-07

**Authors:** Zhixiao Xu, Chengshui Chen

**Affiliations:** ^1^Department of Pulmonary and Critical Care Medicine, The First Affiliated Hospital of Wenzhou Medical University, Wenzhou, 325000 Zhejiang, China; ^2^The Interventional Pulmonary Key Laboratory of Zhejiang Province, Wenzhou, 325000 Zhejiang, China

## Abstract

**Background:**

Lung adenocarcinoma (LUAD) is one of the most life-threatening malignancies. The crucial role of bone morphogenetic protein (BMP)/BMP receptors reveals the significance of exploring BMP protein-related prognostic predictors in LUAD.

**Methods:**

The mRNA expression of BMPs/BMP receptors was investigated in LUAD and normal lung tissues. Gene Ontology and the Kyoto Encyclopedia of Genes and Genomes pathway analyses were performed, and the prognostic values were assessed by Kaplan-Meier Plotter. Univariate and multivariate Cox regression analyses were executed to ascertain the correlation between overall survival (OS) and the mRNA expression of BMPs/BMP receptors. The receiver operating characteristic (ROC) curves were implemented to evaluate the predictive power of the prognostic model. Then, the prognostic model was validated in the GEO cohort. Furthermore, a nomogram comprising the prognostic model was established.

**Results:**

The mRNA expression of BMP2/5/6/R2, ACVRL1, and TGFBR2/3 was lower in LUAD tissues than in normal lung tissues. High expression of BMP2/4/5/R1A/R2, ACVR1/2A/L1, and TGFBR1/3 was associated with better OS, while BMP7 and ACVR1C/2B were associated with poorer OS. Three genes (BMP5, BMP7, and ACVR2A) were screened by univariate and multivariate Cox regression analyses to develop the prognostic model in TCGA. Significantly better survival was observed in LUAD patients with a low-risk score than those with a high-risk score. The ROC curves confirmed the good performance of the prognostic model, then, the prognostic model was validated in the GSE31210 dataset. A nomogram was constructed (AUCs>0.7). And hub genes were further evaluated, including gene set enrichment analysis and immune cell infiltration.

**Conclusions:**

BMP5, BMP7, and ACVR2A are potential therapeutic targets in LUAD. The three-gene prognostic model and the nomogram are reliable tools for predicting the OS of LUAD patients.

## 1. Introduction

Lung cancer is the main cause of cancer-related deaths [1]. Non-small cell lung cancer (NSCLC) is the most common type of lung cancer, of which, adenocarcinoma is the main subtype [2, 3]. NSCLC is one of the most successful applications of precision medicine [4]; no doubt investigating new molecular biomarkers or therapeutic targets has become a research trend [5]. Numerous novel biomarkers for cancer treatment based on large-scale, genome-wide association research databases have emerged [6], but there is an urgent need to identify novel molecular biomarkers and therapeutic targets for lung adenocarcinoma (LUAD).

Bone morphogenetic protein (BMP), including a large class of signaling molecules, belongs to the transforming growth factor- (TGF-) *β* superfamily whose members are involved in regulating a variety of biological processes [7]. BMPs exert their biological effects through type I and II receptors which are two structurally similar, single transmembrane serine/threonine kinase receptors. Seven type I receptors (activin A receptor-like type 1 (ACVRL1/ALK1), activin receptor A type I (ACVR1/ALK2), bone morphogenetic protein receptor type 1A (BMPR1A/ALK3), ACVR1B/ALK4, transforming growth factor-beta receptor 1 (TGFBR1/ALK5), BMPR1B/ALK6, and ACVR1C/ALK7) and five type II receptors (TGFBR2, TGFBR3, BMPR2, ACVR2A, and ACVR2B) have been shown to bind BMPs [8].

Researchers have discovered that BMPs and BMP receptors could affect the prognosis of patients in multiple types of cancer including gastric cancer [7], colorectal cancer [9], and lung cancer [10]. Moreover, the dual role of BMPs in both cancer development and suppression has led BMPs to be regarded as powerful therapeutic targets [11]. However, the underlying functions and mechanisms of BMPs and BMP receptors remain unclear, and a comprehensive mRNA profiling of BMPs and BMP receptors in LUAD has not been performed. The present study involved database research and in-depth bioinformatic analyses to determine the prognostic significance of BMPs and BMP receptors in LUAD.

## 2. Materials and Methods

### 2.1. Data-Mining Analysis by ONCOMINE Database

The online cancer database ONCOMINE (https://www.oncomine.org/) was beneficial to data mining of the transcriptional expression of BMPs/BMP receptors in 20 types of cancer. The student's *t*-test was used to assess whether there was a significant difference between the transcriptional expression of BMPs/BMP receptors in LUAD samples and those in normal lung samples. The *P* < 1*e* − 4 and fold change (FC) > 2 were selected as the cut-off criteria, respectively.

### 2.2. The Relationship between the Abnormal Expression of BMPs/BMP Receptors and the Characteristics of LUAD

The LUAD dataset containing data from 517 cases was from cBioPortal (http://www.cbioportal.org/), an open-access dataset used to sort out multiple cancer genes. The Mann–Whitney (M-W) test was employed to compare the mRNA expression of BMPs/BMP receptors between two groups of LUAD patients with different clinical features. The Kruskal-Wallis (K-W) test was used for multiple group comparison. If the results of the K-W test were significant, a further Dunn's multiple comparison test was conducted.

### 2.3. Gene Ontology and Pathway Enrichment Analysis

The Gene Ontology (GO) analysis was performed to investigate the function of BMPs/BMP receptors using the R package “clusterProfiler” [12]. The Kyoto Encyclopedia of Genes and Genomes (KEGG) pathway analysis was also executed.

### 2.4. Kaplan-Meier Plotter

According to the median gene expression in the online openly available database Kaplan-Meier Plotter (http://www.kmplot.com/), the mRNA transcription level of BMPs/BMP receptors was divided into two groups (high vs. low). Importantly, their prognostic values were investigated, and the hazard ratio (HR) with 95% confidence intervals (CI) and Log-rank *P* values were displayed. *P* value < 0.05 was considered as the cut-off criteria. The Kaplan-Meier (K-M) survival curves were exhibited based on the value of the most detected probe for each BMPs/BMP receptor.

### 2.5. Definition of the Gene-Related Prognostic Model

The mRNA expression profiles and the corresponding clinical information from the LUAD patients were retrieved from The Cancer Genome Atlas (TCGA) database, containing 533 LUAD tissues. After preprocessing (including removal of abnormal values, screening of samples with tumor purity greater than 60%, expression matrix standardization and filtering, and removal of genes with low expression levels), 322 samples were obtained. Univariate and multivariate Cox regression analyses were executed to explore the correlation between overall survival (OS) and the mRNA expression of each BMPs/BMP receptor. In the univariate Cox regression analyses, when the *P* value < 0.05, the gene was considered significant. Then, multivariate Cox regression analyses were facilitated to evaluate the contribution of a gene as an independent prognostic factor for patient survival. A three-gene-based prognosis risk score was built based on a linear combination of the regression coefficient obtained from the multivariate Cox regression model (*β*) multiplied by the mRNA expression level. The optimal cut-off value used to classify patients into low-risk and high-risk groups was identified using X-tile software. The 322 LUAD patients with survival data were separated into low-risk and high-risk groups based on the optimal cut-off value. The K-M survival curves of low-risk or high-risk cases were exhibited. Time-dependent receiver operating characteristic (ROC) curves were used to evaluate the predictive power of the prognostic model. Then, the prognostic model was validated in the Gene Expression Omnibus (GEO) dataset (GSE31210).

### 2.6. Building and Validating a Predictive Nomogram

Univariate and multivariate Cox regression analyses were implemented, with the clinical traits as independent variables and the OS as the dependent variable. All reported *P* values were two-sided, and the HR and 95% CI were displayed. In this study, a combined model constructed on all independent predictive factors derived from multivariate Cox regression analyses was used to develop a nomogram to evaluate the probability of 1-, 3-, and 5-year OS in LUAD patients. Subsequently, verifications including discrimination and calibration were carried out. The discriminative power of the nomogram was assessed using the concordance index (C-index) through a bootstrap method with 1000 resamples. The value of the C-index fluctuated between 0.5 and 1.0, where the closer the value is to 1.0, the more perfect the ability to correctly distinguish the results of the model. The calibration curve of the nomogram was investigated graphically, and overlapping with the reference line indicated that the model was very suitable. Decision curve analysis (DCA) was conducted to determine the clinical utility of a predictive nomogram. At the same time, ROC curve analyses were performed to compare the predictive accuracy of the combined model with those of a single significant prognostic factor.

### 2.7. Gene Set Enrichment Analysis

Gene set enrichment analysis (GSEA) of a single key gene was conducted using the R package “clusterProfiler” to explore the potential function. The c2.cp.kegg.v7.2.entrez.gmt in the Molecular Signatures Database was used as the reference gene set. The adjusted *P* value < 0.05 was set as the cut-off criterion.

### 2.8. Tumor Immunity Estimation Resource (TIMER) Database Analysis

In the TIMER (https://cistrome.shinyapps.io/timer/), immune cell infiltration was systematically analyzed based on RNA sequencing data of various tumors. The expression levels of BMP5/7 and ACVR2A in LUAD and its correlations with the abundances of six tumor-infiltrating immune cells (TILCs) were analyzed through the corresponding functional modules. The six TILCs included B cells, CD4^+^ T cells, CD8^+^ T cells, macrophages, neutrophils, and dendritic cells. Spearman's correlation coefficient and *P* value were calculated, and the gene expression levels were presented as log 2 RSEM values.

## 3. Results

### 3.1. Transcriptional Levels of BMPs/BMP Receptors in LUAD Patients

The BMPs/BMP receptors transcriptional level was compared between various cancers and para-carcinoma tissues based on the ONCOMINE database. BMPs/BMP receptors were generally downexpressed in most tumors as presented in Figure S1. Most BMPs/BMP receptors were downregulated in lung cancer tissues, except BMP7, ACVR1C, and ACVR2B which were upregulated in one dataset. This might be due to the limited number of samples. The mRNA level of all BMPs/BMP receptors significantly decreased in LUAD tissues (Table 1).

### 3.2. The Association of BMPs/BMP Receptors mRNA Expression and the Clinical Features of LUAD Patients

**Figure 1:**
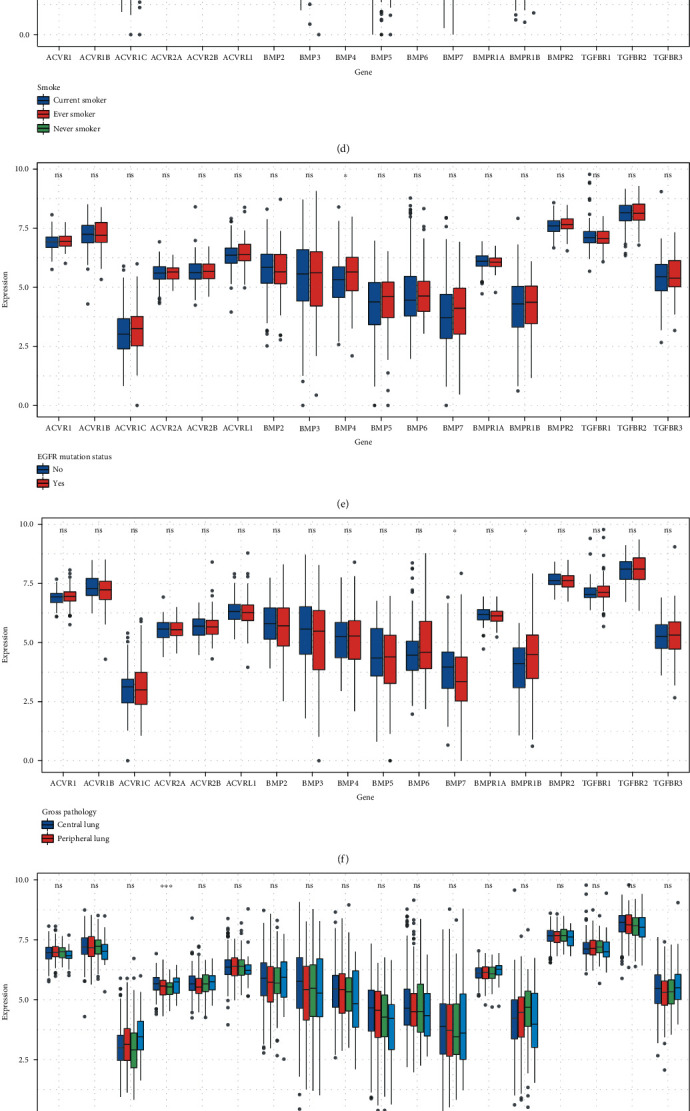
Changes in BMPs/BMP receptors expression according to various clinical characteristics. Differentially expressed BMPs/BMP receptors according to age (a), sex (b), race (c), smoke (d), EGFR mutation (e), gross pathology (f), stage (g), tumor size (h), lymph node metastasis (i), and distant metastasis (j). BMP: bone morphogenetic protein. Note: ns: *P* > 0.05; ∗: *P* ≤ 0.05; ∗∗: *P* ≤ 0.01; ∗∗∗: *P* ≤ 0.001; ∗∗∗∗: *P* ≤ 0.0001.

The relationships between the mRNA expression of BMPs/BMP receptors and the clinical characteristics of LUAD patients were evaluated showing no significant difference between <65 and ≥65 groups, except for TGFBR2/3, BMPR2, and ACVR2A/2B, with only ACVR2B reduced in the older group (Figure 1(a)). A significant difference in BMP3/6/7, ACVRL1, and TGFBR2/3 expression was observed across gender (Figure 1(b)). BMP4, BMPR1A/2, ACVR1B, and TGFBR1 exhibited lower expression in White than in non-White individuals (Figure 1(c)). Interestingly, only the mRNA expression of BMP4 was related to the EGFR mutation (Figure 1(d)).

The mRNA expression of BMP7 (*P* = 0.0260) was significantly increased in the central lung group, while BMPR1B (*P* = 0.0110) mRNA expression was significantly increased in the peripheral lung group (Figure 1(f)). Upregulated ACVR1C and ACVR2A/2B were also associated with distant metastasis (Figure 1(j)). Lymph node status had a relationship with BMPR1B and ACVR2A/2B mRNA expression (Figure 1(i)).

The size of the tumor also affected the expression of BMP3 (*P* = 0.0047), BMP7 (*P* = 0.0210), BMPR1B (*P* = 0.0460), ACVR2A (*P* = 0.0350), ACVRL1 (*P* = 0.0045), TGFBR2 (*P* = 0.0170), and TGFBR3 (*P* = 0.0150) according to the K-W test (Figure 1(h)). Dunn's multiple comparisons tests revealed increased BMP3 (*P* = 0.0037), BMP7 (*P* = 0.0081), ACVR2A (*P* = 0.0071), ACVRL1 (*P* = 0.0012), TGFBR2 (*P* = 0.0139), and TGFBR3 (*P* = 0.0037) expression in the T1 group compared to the T2 group, and Dunn's multiple comparisons tests revealed increased BMP3 (*P* = 0.0285), and ACVR2A (*P* = 0.0116) expression in the T1 group compared to the T3 group. Dunn's multiple comparisons tests also revealed decreased BMPR1B (*P* = 0.0180) and increased ACVR2A (*P* = 0.0106) expression in the T1 group compared to the T4 group.

BMP2/3/4/7, ACVR1/2A/2B, and TGFBR2/3 were related to smoking, with the Dunn's multiple comparison test showing that the mRNA expression of BMP2 (*P* = 0.0187), ACVR1 (*P* = 0.0178), ACVR2B (*P* = 0.0018), and TGFBR2 (*P* = 0.0007) were higher in the current smoker group compared with the ever-smoker group. The expression of BMP3 (*P* = 0.0001), BMP4 (*P* = 0.0204), ACVR2A (*P* = 0.0143), ACVR2B (*P* = 0.0020), and TGFBR2 (*P* = 0.0026) were significantly different between the never-smoker group and the current-smoker group. The mRNA expression of BMP3 (*P* = 0.0020) and BMP7 (*P* = 0.0085) was different between the never-smoker group and the ever-smoker group. Interestingly, TGFBR3 was significantly different in pairwise comparisons.

### 3.3. Functional Annotation of the BMPs/BMP Receptors

**Figure 2:**
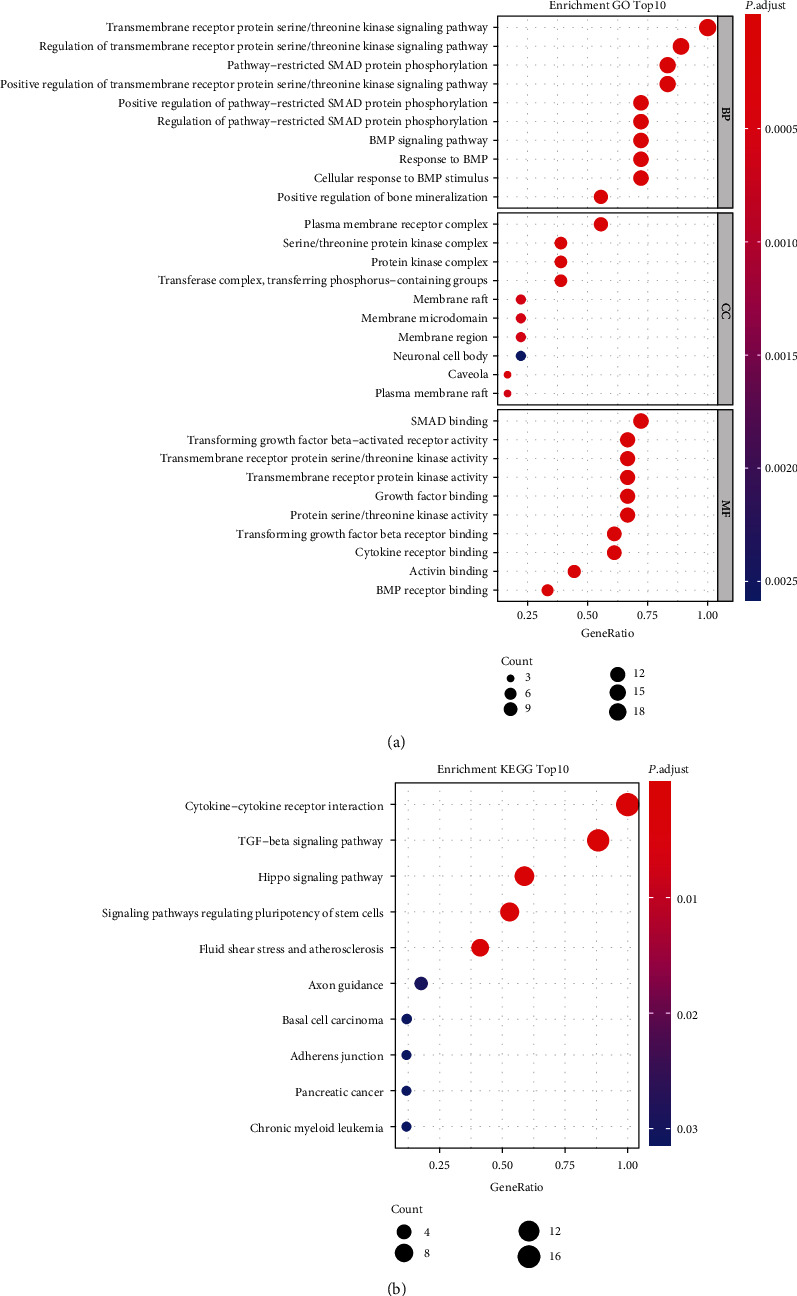
The functional annotation of BMPs/BMP receptors. (a) The Gene Ontology analysis according to the biological process, cellular component, and molecular function. (b) The Kyoto Encyclopedia of Genes and Genomes analysis of BMPs/BMP receptors. BMP: bone morphogenetic protein.

The GO analysis showed that changes in the BP were significantly enriched in the transmembrane receptor protein serine/threonine kinase signaling pathway and its related regulation and restricted Smad protein phosphorylation and its related regulation (Figure 2(a)). For the CC, these genes were enriched in the plasma membrane receptor complex, serine/threonine kinase complex, protein kinase complex, transferase complex, and transferring phosphorus-containing groups (Figure 2(a)). Variations in MF were mainly enriched in Smad binding, TGF-*β* activated receptor binding, and transmembrane receptor protein serine/threonine kinase activity (Figure 2(a)). The KEGG pathway analysis revealed that the TGF-*β* signaling pathway, cytokine-cytokine receptor interaction, and Hippo signaling pathway were associated with the genes (Figure 2(b)).

### 3.4. The Prognostic Significance of BMPs/BMP Receptors in LUAD Patients

The prognostic significance of the mRNA expression of BMPs/BMP receptors in LUAD patients was determined through the Kaplan-Meier plotter, revealing that 12 BMPs/BMP receptors were significantly associated with OS. High expression of BMP2, BMP4, BMP5, BMPR1A, BMPR2, ACVR1, ACVR2A, ACVRL1, and TGFBR3 was associated with better OS, while BMP7, ACVR1B, and TGFBR2 were associated with poorer OS (Table 2).

### 3.5. A Good Performance for the Prognostic Model in Predicting the OS of LUAD Patients

Univariate Cox regression was performed in the TCGA to ascertain the correlation between differentially expressed genes and OS of LUAD patients and identified three genes that were significantly related to the OS of LUAD patients when the *P* value was < 0.05. Then, a multivariate Cox regression analysis was implemented after preliminary filtering, and finally, three genes were selected to establish a prediction model. The predictive model was characterized by the linear combination of the expression levels of the three genes weighted by their relative coefficient from the multivariate Cox regression as follows:

risk score = (−0.12332∗mRNA expression level of BMP5) + (0.08118∗mRNA expression level of BMP7) + (−0.35362∗mRNA expression level of ACVR2A).

**Figure 3:**
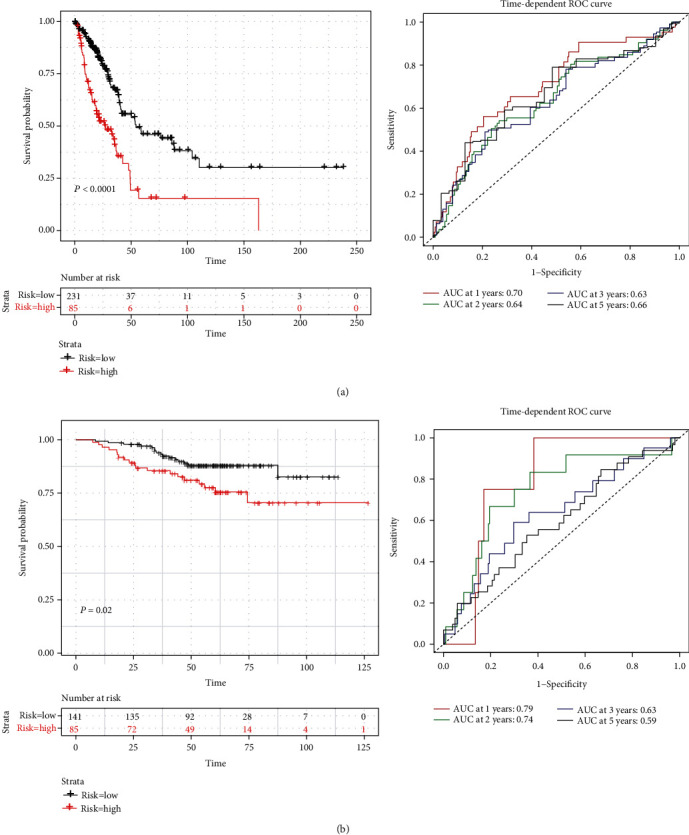
K-M and time-dependent ROC curves for the prognostic model in the TCGA-LUAD cohort (a) and the GEO-LUAD cohort (b). The K-M survival curves showed the overall survival based on the relatively high- and low-risk patients classified by the optimal cut-off value. The time-dependent ROC curve analyses of survival prediction by the prognostic model. LUAD: lung adenocarcinoma; ROC: receiver operating characteristic.

In this study, for the 316 patients with survival time, the three-gene expression risk score was calculated, and the X-tile was used to obtain the optimal cut-off value of the risk score (Figure S2). A total of 83 patients were classified as high-risk patients because their risk scores were higher than the cut-off value, while the other 233 patients were assigned to the low-risk group (Figure S3). Based on the three genes, the K-M curves of the two groups were significantly different (*P* < 0.0001; Figure 3(a)). The prognostic capacity of the three-gene signature was estimated by calculating the AUCs of the time-dependent ROC curves (Figure 3(a)), which were 0.70, 0.64, 0.63, and 0.66 for the 1-, 2-, 3-, and 5-year survival times, respectively, indicating that the prognostic model had high sensitivity and specificity (Figure 3(a)).

To assess the predictive value of the prognostic model in predicting the OS of LUAD patients in other datasets, the prognostic model was evaluated in the GSE31210 cohort. A total of 226 patients were categorized into the low-risk group (*n* = 148) and high-risk group (*n* = 78) using the optimal risk cut-off value of the GEO cohort (the same as the risk scoring model of the TCGA cohort) (Figure S2, S3). Consistent with the results in the TCGA, the OS of LUAD patients in the GSE31210 of the high-risk group was significantly higher than that of the low-risk group (*P* = 0.02; Figure 3(b)). In addition, the time-dependent ROC curves on the survival prediction of the prognostic model demonstrated that the AUC at 1-year was 0.79, at 2-years was 0.74, at 3-years was 0.63, and at 5 years was 0.59, indicating that the prognostic model could predict the OS of LUAD patients (Figure 3(b)).

### 3.6. Construction of a Predictive Nomogram

**Figure 4:**
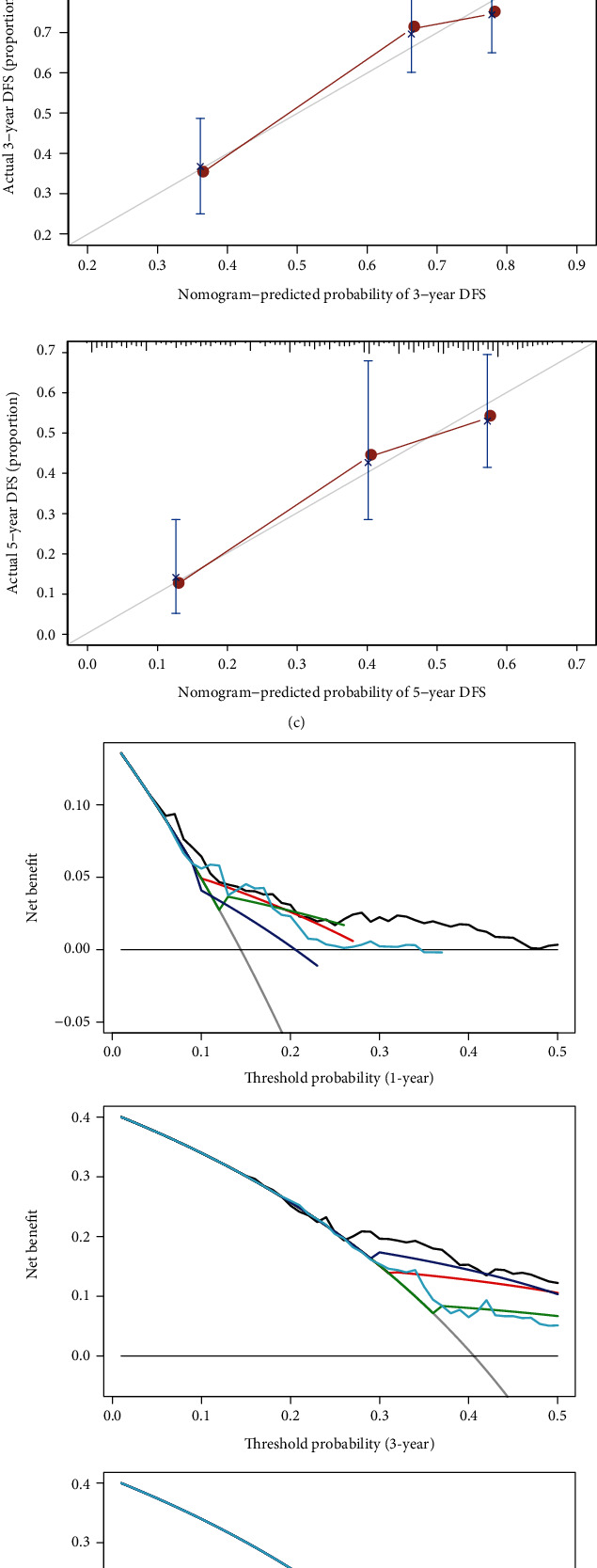
The nomogram to anticipate prognostic probabilities in LUAD. (a) Univariate and multivariate association of the three-gene prognostic model and clinical characteristics with overall survival. Red represented statistical significance, and blue represented no statistical significance. (b) Nomogram predicting 1-, 3-, and 5-year OS for LUAD patients. The nomogram was applied by adding up the points identified on the point scale of each variable. The total points projected on the bottom scales indicate the probability of 1-, 3-, and 5-year OS. (c) The calibration curve for predicting 1-, 3-, and 5-y OS of LUAD patients. (d) The DCA curves could intuitively evaluate the clinical benefit of the nomogram and the scope of application of the nomogram to obtain clinical benefits. (e) Time-dependent ROC curve analysis evaluated the accuracy of the nomograms. LUAD: lung adenocarcinoma; OS: overall survival; DCA: decision curve analysis.

The predictive model with the univariate Cox regression analysis, clinical covariates of pathologic stage, tumor size, and lymph node metastasis had some predictive value; however, sex, age, race, EGFR mutation, gross pathology, and distant metastasis did not correlate with OS (Figure 4(a)). Therefore, the pathologic stage, tumor size, lymph node metastasis, and the three-gene prognostic model (risk score) were incorporated into the multivariate Cox regression analysis, showing that they were independent prognostic factors associated with OS (Figure 4(a)).

To establish a clinically applicable method for predicting the survival probability of LUAD patients, a nomogram was developed to predict the probability of the 1-, 3-, and 5-year OS in the TCGA cohort. The predictors of the nomogram included four independent prognostic factors (pathologic stage, tumor size, lymph node metastasis, and the three-gene prognostic model; Figure 4(b)). The C-index for the model for evaluation of OS was 0.6696159, with 1000 cycles of bootstrapping, bias-corrected-C-index was 0.6771448. The calibration plots illustrated that the nomogram performed well based on the principle that the 45° line represented the best prediction (Figure 4(c)). DCA evaluates clinical usefulness, showing that the nomogram exhibited a good net benefit (Figure 4(d)). Also, compared with the pathologic stage, tumor size, lymph node metastasis, and the three-gene prognostic model, the AUCs of the nomogram were the largest (AUCs > 0.7) (Figure 4(e)), confirming that the nomogram was a good tool for predicting survival for patients with LUAD, which might be a benefit for patient consultation, decision-making, and follow-up schedule.

### 3.7. Gene Set Enrichment Analysis of BMP5/7 and ACVR2A.

**Figure 5:**
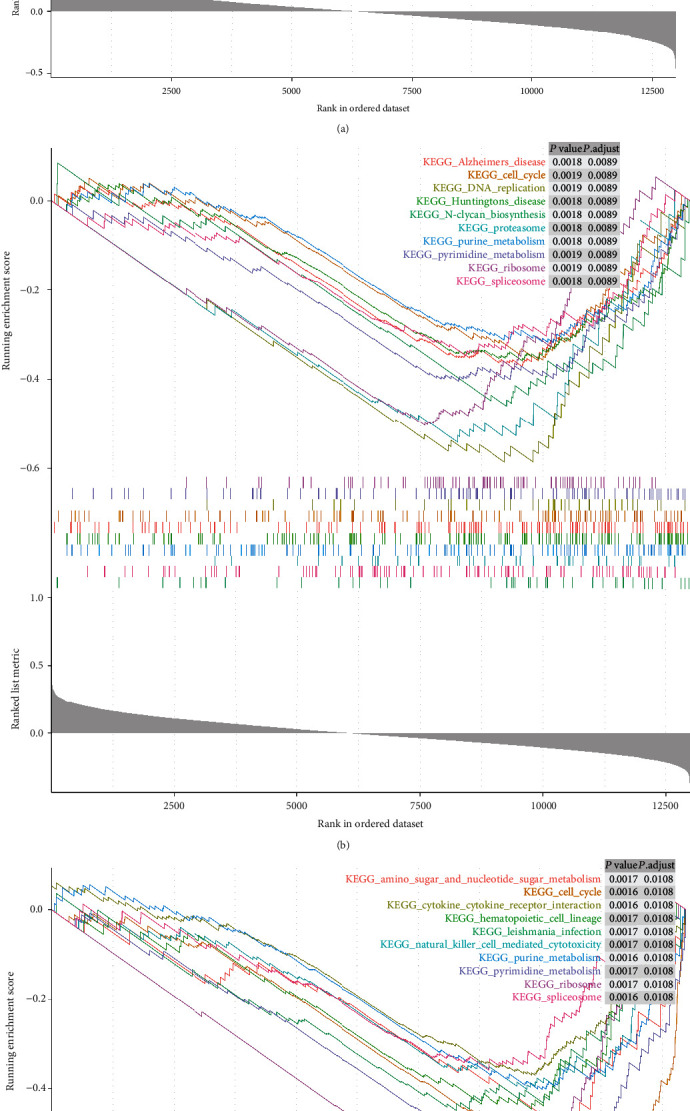
Gene set enrichment analysis (GSEA) of BMP5 (a), BMP7 (b), and ACVR2A (c) showing the Kyoto Encyclopedia of Genes and Genomes (KEGG) pathways. BMP: bone morphogenetic protein; ACVR2A: activin receptor A type IIA.

The GSEA analysis showed that the low expression of ACVR2A and BMP7 was enriched in “cell cycle,” “purine metabolism,” “pyrimidine metabolism,” “ribosome,” and “spliceosome.” The “cytokine-cytokine receptor interaction” was enriched in the high expression levels of BMP5 and the low expression of ACVR2A (Figure 5).

BMP5, BMP7, and ACVR2A were involved in DNA proliferation, purine and pyrimidine nucleoside biosynthesis, purine and pyrimidine metabolism. Moreover, there was a close relationship between cytokines and BMP5/7, ACVR2A.

### 3.8. The Expression of BMP5/7 and ACVR2A Positively Correlated with Infiltrating Immune Cells in LUAD.

**Figure 6:**
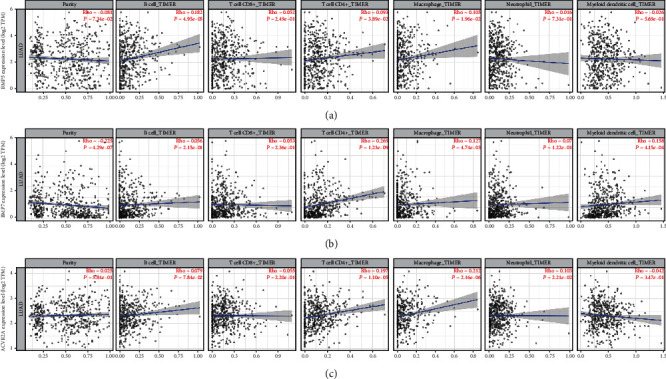
Association of BMP5 (a), BMP7 (b), and ACVR2A (c) expression with immune cell infiltration in LUAD. BMP: bone morphogenetic protein; ACVR2A: activin receptor A type IIA; LUAD: lung adenocarcinoma.

TILCs are associated with sentinel lymph node metastasis and prognosis of tumors, and tumor purity impacts immune infiltration in the analysis of clinical sample data based on genetic testing. The BMP5/7 expression significantly negatively correlated with tumor purity, while ACVR2A had no significant correlation with tumor purity (Figure 6). Interestingly, there was a significant positive correlation between all three genes and the level of infiltrating CD4^+^ T cells and macrophages (Figure 6). These findings strongly demonstrated that BMP5/7 and ACVR2A could recruit immune cells in the tumor microenvironment (TME) in LUAD, especially CD4^+^ T cells and macrophages.

## 4. Discussion

The present study sought to explore the involvement of BMPs in LUAD by analyzing publicly available data, specifically focusing on their prognostic value. Three hub genes, BMP5, BMP7, and ACVR2A, were identified, and their mRNA expression was relevant to the prognosis value in LUAD patients using the TCGA-LUAD database. These three hub genes were used to construct a prognostic model, which was further validated in another GEO database (GSE31210). Furthermore, a practical nomogram was developed.

BMP2 was downregulated in LUAD samples compared to normal lung samples, which was in contrast to the overexpression of BMP2 previously reported in NSCLC samples [22, 23]. This difference may be due to NSCLC including LUAD and lung squamous cell carcinoma (LUSC). The high expression of BMP2 in primary lung fibroblasts could predict a poor prognosis of LUAD patients [24]. BMP2 silencing inhibited the proliferation and migration of lung cancer cells [25], and BMP2 signaling activation promoted bone metastases and invasion in NSCLC [26]. There was no difference in the expression of BMP4 in LUAD tissue compared with normal lung tissue but it was associated with the ethnicity and prognosis of LUAD in the present study. BMP4 was overexpressed in NSCLC samples, and the NSCLC patients with high BMP4 levels had significantly shorter progression-free survival (PFS) and OS [27]. Previous studies have shown that the expression of BMP4 was positively correlated with the widespread metastasis of human tumors [28]. BMP3 and BMP6 mRNA expression is downregulated in NSCLC tissues and cell lines, which is related to the development of lung tumors [29, 30], and the expression of BMP6 was also downregulated in LUAD tissues in the present study. BMP6 was a tumor suppressor of lung cancer, and the loss of BMP6 expression was tightly associated with the poor prognosis of the NSCLC patients [31]. Based on our results, BMPR1A/2 expression had a prognostic value in LUAD patients. Furthermore, BMPR1A, BMPR1B, and BMPR2 were associated with clinical factors, including age, ethnicity, tumor size, and lymph node metastasis. BMP7 signaling via BMPR1A and BMPR1B could inhibit the proliferation of lung cancer cells [32]. ACVR1 correlated with better OS in our study but should be regarded as a complex regulator of cancer biology, because ACVR1 could act as a tumor suppressor or oncogene, depending on the type of cancer or cell, or ligand involved [33]. The ACVR1B gene was correlated with the risk of lung cancer in adults exposed to environmental tobacco smoke [34]; similarly, ACVR1B was associated with poor prognosis in the present study.

The expression of TGFBR2 in NSCLC tissue samples was low compared to adjacent normal lung tissue [35], and the expression of TGFBR3 in NSCLC was lower than that in normal lung tissue [36]. We observed similar results in LUAD tissues. Moreover, the expression of TGFBR3 was downregulated in the blood and tumor tissue cells of patients with stage I LUAD [37]. The high expression of TGFBR2 was an important risk factor for the diminishing of OS and disease-free survival (DFS) in NSCLC patients [38], and the *P* value of the prognostic value of TGFBR2 based on the Kaplan-Meier Plotter was also <0.05, which suggests that there was a tight relationship between the expression of TGFBR2 and the prognosis of LUAD patients. Regulating TGFBR2 signaling could induce multidrug resistance in LUAD [39]. Decreased expression of TGFBR2 in human NSCLC was related to more invasive tumor behavior and inflammation, and the deletion of TGFBR2 in mouse airway epithelial cells promoted the formation of adenocarcinoma [40]. Global profile analysis of LUAD identified TGFBR2 as an aggressive inhibitor [41].

The mRNA and protein expression of BMP5 in LUAD tissue were remarkably high compared with lung squamous cell carcinoma tissue [42]. Our bioinformatics analysis also revealed decreased expression of BMP5 in LUAD tissue compared to normal lung tissue. Besides, downregulated BMP5 correlated with poor prognosis. It had been illustrated that epithelial-mesenchymal transition induced by TGF-*β* was mediated by Blimp-1-dependent inhibition of BMP5 [43]. PinX1-arrested cell cycle transition led to the NSCLC cell proliferation, and BMP5 might take part in PinX1-associated cell proliferation and cell cycle transition [44]. Although BMP5 may play a role in LUAD, the underlying mechanisms have not been fully elucidated. The GSEA analysis revealed that BMP5 was involved in the calcium signaling pathway, cytokine-cytokine receptor interaction, TGF-*β* signaling pathway, gonadotropin-releasing hormone (GnRH) signaling pathway, and mitogen-activated protein kinase (MAPK) signaling pathway. The GnRH signaling pathway not only plays an important role in human development but also in the occurrence and development of human cancer [45, 46]. Also, previous studies have shown that the MAPK signaling pathway plays a vital role in preventing the occurrence and development of cancer [47]. These candidate pathways mentioned above demanded further experimental verification and could be clues of future mechanism studies.

It has been shown that BMP7 not only promoted growth stimulatory but also promoted inhibitory effects in tumor cells [48–50], and BMP7 was related to the postoperative prognosis of NSCLC patients [51]. In this study, we discovered that the expression level of BMP7 in LUAD tissues was not significantly different from that in adjacent noncancerous tissues, but upregulated BMP7 correlated with poor prognosis, female, central lung cancer, and lower T stage in LUAD patients, indicating that BMP7 had a close relationship with LUAD. Previous studies have shown that the expression of BMP7 was related to lymph node metastasis in patients with lung cancer [48]. Moreover, BMP7 expression in NSCLC was correlated with the progression of the tumor and poor prognosis [51]. Correspondingly, the GSEA analysis showed that the low expression of BMP7 was enriched in the cell cycle, DNA replication, proteasome, purine metabolism, and pyrimidine metabolism. When BMP7 binds to BMPRII, it then binds to BMPRI. Restricted Smads (R-Smads) include Smad1/5/8, also known as Smad1/5/9, are phosphorylated, and then enter the nucleus with Smad4, where they regulate target genes [52]. BMP7 was regarded as an effective target for inhibiting cell growth and inducing cell apoptosis [53, 54]. Several reports have demonstrated that BMP7 promoted cell invasiveness and motility of lung cancer cells [42, 55]. The miR-137/BMP7 axis could contribute to the progression of NSCLC [56].

In the present study, upregulated ACVR2A correlated with poor prognosis in LUAD patients, and the GSEA analysis showed that the low expression of ACVR2A was enriched in the cell cycle, cytokine-cytokine receptor interaction, purine metabolism, and pyrimidine metabolism. Activin A could inhibit the transmission of BMP signals through combining with ACVR2A and ACVR2B [8]. Unfortunately, few studies have focused on the role of ACVR2A in LUAD.

Gene-based risk assessment models for lung cancer are emerging [57, 58]. In the present study, the three hub genes (BMP5, BMP7, and ACVR2A) were used to construct a prognostic model. The GSE31210 cohort was used to validate the prognostic model predictive power. Fortunately, for both the TCGA and GEO cohorts, the AUCs of the ROC curves for the prognostic model indicated that the three-gene signature had a good performance for survival prediction. We also developed a nomogram to accurately predict the likelihood of OS in LUAD patients. The calibration plots demonstrated that actual survival was closely related to predicted survival, confirming good prediction performance. At the same time, we proved that the nomogram was effective in terms of C-index, AUC, and DCA. Overall, the BMPs/BMP receptors prognostic signature accurately predicted survival outcomes of LUAD patients in our study, thereby has potential for clinical applications.

However, several limitations should be considered. First, the information in the TCGA database was limited, for example, there were 218 patients with missing information about radiotherapy. Second, our nomogram was not externally verified in the GEO database because GSE31210 lacked clinical data. A robust nomogram should be verified externally in different cohorts; therefore, the nomogram needs to be further verified in multicenter clinical trials and prospective studies. In the future, we will also explore the possibility of incorporating more prognostic variables and other regression modeling methods to further improve performance and prediction accuracy.

## 5. Conclusions

In conclusion, the expression of BMPs/BMP receptors in LUAD was different from normal lung tissues, and the high expression of most of them was associated with poor prognosis in LUAD patients. The three-gene (BMP5, BMP7, and ACVR2A) prognostic model performed well in predicting the OS of LUAD patients. And the nomogram comprising the prognostic model was a reliable tool for predicting the OS of LUAD patients. BMP5, BMP7, and ACVR2A are potential therapeutic targets for LUAD.

## Figures and Tables

**Table 1:** Differential expression of BMPs/BMP receptors in lung adenocarcinoma and normal lung tissue from the ONCOMINE database.

	Fold change	*P* value	*t*-test	Ref
BMP2	-10.392	2.32*E*-07	-6.654	Bhattacharjee et al. [13]
	-2.455	1.48*E*-08	-6.567	Su et al. [14]
	-2.539	1.11*E*-14	-8.858	Landi et al. [15]
	-2.621	4.01*E*-05	-4.433	Stearman et al. [16]
	-4.144	2.05*E*-11	-7.955	Hou et al. [17]
BMP5	-6.923	5.58*E*-08	-6.680	Beer et al. [18]
	-2.717	2.50*E*-12	-9.058	Okayama et al. [19]
	-2.898	5.07*E*-06	-5.034	Su et al. [14]
	-3.267	7.37*E*-12	-8.133	Hou et al. [17]
BMP6	-2.544	2.69*E*-10	-9.409	Okayama et al. [19]
BMPR2	-2.281	5.66*E*-17	-10.377	Hou et al. [17]
	-2.038	1.77*E*-10	-10.537	Okayama et al. [19]
ACVRL1	-7.776	4.99*E*-09	-8.992	Bhattacharjee et al. [13]
	-4.256	1.08*E*-10	-9.561	Stearman et al. [16]
	-3.295	5.90*E*-32	-18.170	Selamat et al. [20]
	-4.102	4.85*E*-19	-12.206	Hou et al. [17]
	-2.872	3.00*E*-09	-7.082	Su et al. [14]
	-3.827	4.94*E*-12	-12.123	Okayama et al. [19]
TGFBR2	-3.412	1.46*E*-25	-14.922	Selamat et al. [20]
	-2.436	4.17*E*-05	-6.220	Garber et al. [21]
	-2.194	4.18*E*-08	-6.590	Okayama et al. [19]
TGFBR3	-32.278	7.23*E*-11	-11.303	Bhattacharjee et al. [13]
	-5.355	2.25*E*-34	-18.633	Landi et al. [15]
	-4.558	2.62*E*-14	-10.829	Su et al. [14]
	-4.344	4.41*E*-24	-18.553	Okayama et al. [19]
	-4.606	2.16*E*-37	-19.117	Selamat et al. [20]
	-6.808	4.83*E*-12	-9.541	Beer et al. [18]
	-6.330	3.34*E*-20	-12.985	Hou et al. [17]
	-6.154	6.78*E*-08	-7.451	Stearman et al. [16]

**Table 2:** Association of BMPs/BMP receptors expression with prognosis in LUAD patients revealed by Kaplan-Meier Plotter.

Name	Cut-off value (counts)	Range (counts)	*P* value	HR	No. patients
BMP2	337	5–7330	0.0002	0.64 (0.51-0.81)	719
BMP3	49	1–1053	0.1001	0.82 (0.65-1.04)	719
BMP4	26	2 –5308	0.0239	0.76 (0.60-0.96)	719
BMP5	86	0–4234	6.90E-09	0.50 (0.39-0.63)	719
BMP6	185	4–8180	0.9815	1.00 (0.79-1.26)	719
BMP7	156	1–3030	0.2600	1.17 (0.89-1.54)	719
BMPR1A	370	6–1789	1.60E-08	0.50 (0.39-0.64)	719
BMPR1B	42	-1–5621	0.2820	0.85 (0.62-1.15)	672
BMPR2	752	-1–9643	0.0444	0.75 (0.56-0.99)	672
ACVR1	1283	138–4666	1.20E-07	0.54 (0.42-0.68)	719
ACVR1B	307	5–2312	0.0015	1.45 (1.15-1.83)	719
ACVR1C	50	-1–2919	0.4622	1.11 (0.85-1.44)	672
ACVR2A	168	-1–1710	0.0007	0.64 (0.49-0.83)	672
ACVR2B	8	-1–382	0.7751	1.04 (0.80-1.35)	672
ACVRL1	133	-1–3761	0.0158	0.64 (0.44-0.92)	672
TGFBR1	1212	-1–13618	0.1066	0.80 (0.61-1.05)	672
TGFBR2	41	1–658	0.0377	1.28 (1.01-1.63)	719
TGFBR3	268	-1–12138	0.0003	0.61 (0.47-0.80)	672

## Data Availability

The data sets analyzed during this study are publicly available databases, such as The Cancer Genome Atlas (TCGA) database and Gene Expression Omnibus (GEO) dataset.

## Supplementary Materials

Supplementary Materials Figure S1: significantly changed BMPs/BMP receptors expression in different types of cancers. The color was determined by the highest gene rank percentile genes based on fold-change; red illustrated up-regulation and blue indicated down-regulation. BMP: bone morphogenetic protein. Figure S2: X-tile was used to determine the best cutoff value of risk scores in TCGA (a) and GEO (b). The cut-point was shown on a histogram of the entire cohort (middle panels). A Kaplan-Meier plot (right panels; the low subset = gray, the high subset = cyan). Figure S3: the risk scores of the three genes. From top to bottom, the distribution of risk scores, the survival status of each patient, and the heat map of mRNA expression levels of the three genes were displayed for TCGA (a) and GEO (b) database.

## References

[1] Bray F., Ferlay J., Soerjomataram I., Siegel R. L., Torre L. A., Jemal A. (2018). Global cancer statistics 2018: GLOBOCAN estimates of incidence and mortality worldwide for 36 cancers in 185 countries. *CA: a Cancer Journal for Clinicians*.

[2] Barlesi F., Mazieres J., Merlio J. P. (2016). Routine molecular profiling of patients with advanced non-small-cell lung cancer: results of a 1-year nationwide programme of the French Cooperative Thoracic Intergroup (IFCT). *Lancet (London, England)*.

[3] Travis W. D., Brambilla E., Nicholson A. G. (2015). The 2015 World Health Organization classification of lung tumors: impact of genetic, clinical and radiologic advances since the 2004 classification. *Journal of thoracic oncology*.

[4] Yang C. Y., Yang J. C., Yang P. C. (2020). Precision management of advanced non-small cell lung cancer. *Annual Review of Medicine*.

[5] Tsao A. S., Scagliotti G. V., Bunn P. A. (2016). Scientific advances in lung cancer 2015. *Journal of Thoracic Oncology*.

[6] Dirks R. A., Stunnenberg H. G., Marks H. (2016). Genome-wide epigenomic profiling for biomarker discovery. *Clinical Epigenetics*.

[7] Sun Z., Liu C., Jiang W. G., Ye L. (2020). Deregulated bone morphogenetic proteins and their receptors are associated with disease progression of gastric cancer. *Computational and Structural Biotechnology Journal*.

[8] Olsen O. E., Wader K. F., Hella H. (2015). Activin A inhibits BMP-signaling by binding ACVR2A and ACVR2B. *Cell communication and signaling*.

[9] Kodach L. L., Wiercinska E., de Miranda N. F. (2008). The bone morphogenetic protein pathway is inactivated in the majority of sporadic colorectal cancers. *Gastroenterology*.

[10] Bieniasz M., Oszajca K., Eusebio M., Kordiak J., Bartkowiak J., Szemraj J. (2009). The positive correlation between gene expression of the two angiogenic factors: VEGF and BMP-2 in lung cancer patients. *Lung cancer (Amsterdam, Netherlands)*.

[11] Bach D. H., Park H. J., Lee S. K. (2017). The dual role of bone morphogenetic proteins in cancer. *Molecular therapy oncolytics*.

[12] Yu G., Wang L.-G., Han Y., He Q. Y. (2012). clusterProfiler: an R package for comparing biological themes among gene clusters. *OMICS: A Journal of Integrative Biology*.

[13] Bhattacharjee A., Richards W. G., Staunton J. (2001). Classification of human lung carcinomas by mRNA expression profiling reveals distinct adenocarcinoma subclasses. *Proceedings of the National Academy of Sciences of the United States of America*.

[14] Su L. J., Chang C. W., Wu Y. C. (2007). Selection of DDX5 as a novel internal control for Q-RT-PCR from microarray data using a block bootstrap re-sampling scheme. *BMC Genomics*.

[15] Landi M. T., Dracheva T., Rotunno M. (2008). Gene expression signature of cigarette smoking and its role in lung adenocarcinoma development and survival. *PLoS One*.

[16] Stearman R. S., Dwyer-Nield L., Zerbe L. (2005). Analysis of orthologous gene expression between human pulmonary adenocarcinoma and a carcinogen-induced murine model. *The American Journal of Pathology*.

[17] Hou J., Aerts J., den Hamer B. (2010). Gene expression-based classification of non-small cell lung carcinomas and survival prediction. *PLoS One*.

[18] Beer D. G., Kardia S. L., Huang C. C. (2002). Gene-expression profiles predict survival of patients with lung adenocarcinoma. *Nature Medicine*.

[19] Okayama H., Kohno T., Ishii Y. (2012). Identification of genes upregulated in ALK-positive and EGFR/KRAS/ALK-negative lung adenocarcinomas. *Cancer Research*.

[20] Selamat S. A., Chung B. S., Girard L. (2012). Genome-scale analysis of DNA methylation in lung adenocarcinoma and integration with mRNA expression. *Genome Research*.

[21] Garber M. E., Troyanskaya O. G., Schluens K. (2001). Diversity of gene expression in adenocarcinoma of the lung. *Proceedings of the National Academy of Sciences of the United States of America*.

[22] Choi Y. J., Kim S. T., Park K. H. (2012). The serum bone morphogenetic protein-2 level in non-small-cell lung cancer patients. *Medical oncology (Northwood, London, England)*.

[23] Fei Z. H., Yao C. Y., Yang X. L., Huang X. E., Ma S. L. (2013). Serum BMP-2 up-regulation as an indicator of poor survival in advanced non-small cell lung cancer patients. *Asian Pacific Journal of Cancer Prevention*.

[24] Rajski M., Saaf A., Buess M. (2015). BMP2 response pattern in human lung fibroblasts predicts outcome in lung adenocarcinomas. *BMC Medical Genomics*.

[25] Chu H., Luo H., Wang H. (2014). Silencing BMP-2 expression inhibits A549 and H460 cell proliferation and migration. *Diagnostic Pathology*.

[26] Huang F., Cao Y., Wu G. (2020). BMP2 signalling activation enhances bone metastases of non-small cell lung cancer. *Journal of Cellular and Molecular Medicine*.

[27] Ju F. J., Meng F. Q., Hu H. L., Liu J. (2019). Association between BMP4 expression and pathology, CT characteristics and prognosis of non-small cell lung cancer. *European Review for Medical and Pharmacological Sciences*.

[28] Kestens C., Siersema P. D., Offerhaus G. J., van Baal J. W. (2016). Correction: BMP4 signaling is able to induce an epithelial-mesenchymal transition-like phenotype in Barrett's esophagus and esophageal adenocarcinoma through induction of SNAIL2. *PLoS One*.

[29] Baylin S. B., Herman J. G., Graff J. R., Vertino P. M., Issa J. P. (1997). Alterations in DNA methylation: a fundamental aspect of neoplasia. *Advances in Cancer Research*.

[30] Dai Z., Popkie A. P., Zhu W. G. (2004). Bone morphogenetic protein 3B silencing in non-small-cell lung cancer. *Oncogene*.

[31] Xiong W., Wang L., Yu F. (2019). Expression of bone morphogenetic protein 6 in non-small cell lung cancer and its significance. *Oncology Letters*.

[32] Xu H., Qi Y., Dun S., Gao Y., Qiu X. (2010). BMP7 signaling via BMPR1A, BMPR1B inhibits the proliferation of lung large carcinoma NCI-H460 cell. *Chinese Journal of Lung Cancer*.

[33] Valer J. A., Sánchez-de-Diego C., Pimenta-Lopes C., Rosa J. L., Ventura F. (2019). ACVR1 function in health and disease. *Cell*.

[34] Spitz M. R., Gorlov I. P., Amos C. I. (2011). Variants in inflammation genes are implicated in risk of lung cancer in never smokers exposed to second-hand smoke. *Cancer Discovery*.

[35] Li G., Wu F., Yang H., Deng X., Yuan Y. (2017). MiR-9-5p promotes cell growth and metastasis in non-small cell lung cancer through the repression of TGFBR2. *Biomedicine & pharmacotherapy = Biomedecine & pharmacotherapie*.

[36] Szymanowska-Narloch A., Jassem E., Skrzypski M. (2013). Molecular profiles of non-small cell lung cancers in cigarette smoking and never-smoking patients. *Advances in Medical Sciences*.

[37] Rotunno M., Hu N., Su H. (2011). A gene expression signature from peripheral whole blood for stage I lung adenocarcinoma. *Cancer prevention research (Philadelphia, Pa)*.

[38] Han Y., Jia C., Cong X. (2015). Increased expression of *TGFβR2* is associated with the clinical outcome of non-small cell lung cancer patients treated with chemotherapy. *PLoS One*.

[39] Li J., Ao J., Li K. (2016). ZNF32 contributes to the induction of multidrug resistance by regulating TGF- *β* receptor 2 signaling in lung adenocarcinoma. *Cell Death & Disease*.

[40] Malkoski S. P., Haeger S. M., Cleaver T. G. (2012). Loss of transforming growth factor beta type II receptor increases aggressive tumor behavior and reduces survival in lung adenocarcinoma and squamous cell carcinoma. *Clinical Cancer Research*.

[41] Borczuk A. C., Kim H. K., Yegen H. A., Friedman R. A., Powell C. A. (2005). Lung adenocarcinoma global profiling identifies type II transforming growth factor-beta receptor as a repressor of invasiveness. *American Journal of Respiratory and Critical Care Medicine*.

[42] Deng T., Lin D., Zhang M. (2015). Differential expression of bone morphogenetic protein 5 in human lung squamous cell carcinoma and adenocarcinoma. *Acta Biochimica et Biophysica Sinica*.

[43] Romagnoli M., Belguise K., Yu Z. (2012). Epithelial-to-mesenchymal transition induced by TGF-*β*1 is mediated by Blimp-1-dependent repression of BMP-5. *Cancer Research*.

[44] Tian X. P., Jin X. H., Li M., Huang W. J., Xie D., Zhang J. X. (2017). The depletion of PinX1 involved in the tumorigenesis of non-small cell lung cancer promotes cell proliferation via p15/cyclin D1 pathway. *Molecular Cancer*.

[45] Gründker C., Emons G. (2017). The role of gonadotropin-releasing hormone in cancer cell proliferation and metastasis. *Frontiers in Endocrinology*.

[46] Harrison G. S., Wierman M. E., Nett T. M., Glode L. M. (2004). Gonadotropin-releasing hormone and its receptor in normal and malignant cells. *Endocrine-Related Cancer*.

[47] Fresco P., Borges F., Diniz C., Marques M. P. (2006). New insights on the anticancer properties of dietary polyphenols. *Medicinal Research Reviews*.

[48] Chen J., Ye L., Xie F., Yang Y., Zhang L., Jiang W. G. (2010). Expression of bone morphogenetic protein 7 in lung cancer and its biological impact on lung cancer cells. *Anticancer Research*.

[49] Duangkumpha K., Techasen A., Loilome W. (2014). BMP-7 blocks the effects of TGF-*β*-induced EMT in cholangiocarcinoma. *Tumor Biology*.

[50] Yeh L. C. (2010). In vitro and in vivo studies on the effects of bone morphogenetic protein-7 on human kidney and lung tumor cells. *International Journal of Biomedical Science*.

[51] Aoki M., Umehara T., Kamimura G. (2019). Expression of bone morphogenetic protein-7 significantly correlates with non-small cell lung cancer progression and prognosis: a retrospective cohort study. *Clinical Medicine Insights Oncology*.

[52] Hogan B. L. (1996). Bone morphogenetic proteins: multifunctional regulators of vertebrate development. *Genes & Development*.

[53] Alarmo E. L., Pärssinen J., Ketolainen J. M., Savinainen K., Karhu R., Kallioniemi A. (2009). BMP7 influences proliferation, migration, and invasion of breast cancer cells. *Cancer Letters*.

[54] Hu M., Cui F., Liu F., Wang J., Wei X., Li Y. (2017). BMP signaling pathways affect differently migration and invasion of esophageal squamous cancer cells. *International Journal of Oncology*.

[55] Liu Y., Chen J., Yang Y., Zhang L., Jiang W. G. (2012). *Μ*olecular impact of bone morphogenetic protein 7, on lung cancer cells and its clinical significance. *International Journal of Molecular Medicine*.

[56] Yang Y. R., Li Y. X., Gao X. Y., Zhao S. S., Zang S. Z., Zhang Z. Q. (2018). Erratum: microRNA-137 inhibits cell migration and invasion by targeting bone morphogenetic protein-7 (BMP7) in non-small cell lung cancer cells. *International Journal of Clinical and Experimental Pathology*.

[57] Liao Y., Wang Y., Cheng M., Huang C., Fan X. (2020). Weighted gene coexpression network analysis of features that control cancer stem cells reveals prognostic biomarkers in lung adenocarcinoma. *Frontiers in Genetics*.

[58] Liao Y., Xiao H., Cheng M., Fan X. (2020). Bioinformatics analysis reveals biomarkers with cancer stem cell characteristics in lung squamous cell carcinoma. *Frontiers in Genetics*.

