# Supplementary material for: Abnormal Expression and Prognostic Significance of Bone Morphogenetic Proteins and Their Receptors in Lung Adenocarcinoma
Source: Biomed Res Int. 2021 May 7;2021:6663990. doi: 10.1155/2021/6663990 (PMC8123996; doi:10.1155/2021/6663990)
Supplement: Supplementary Materials — Figure S1: significantly changed BMPs/BMP receptors expression in different types of cancers. The color was determined by the highest gene rank percentile genes based on fold-change; red illustrated up-regulation and blue indicated down-regulation. BMP: bone morphogenetic protein. Figure S2: X-tile was used to determine the best cutoff value of risk scores in TCGA (a) and GEO (b). The cut-point was shown on a histogram of the entire cohort (middle panels). A Kaplan-Meier plot (right panels; the low subset = gray, the high subset = cyan). Figure S3: the risk scores of the three genes. From top to bottom, the distribution of risk scores, the survival status of each patient, and the heat map of mRNA expression levels of the three genes were displayed for TCGA (a) and GEO (b) database. [file 6663990.f1.zip › FigureS1 (2).pdf]

Analysis Type by Cancer	BMP2 Cancer vs. Normal (red)	BMP2 (blue)	BMP3 Cancer vs. Normal (red)	BMP3 (blue)	BMP4 Cancer vs. Normal (red)	BMP4 (blue)	BMP5 Cancer vs. Normal (red)	BMP5 (blue)	BMP6 Cancer vs. Normal (red)	BMP6 (blue)	BMP7 Cancer vs. Normal (red)	BMP7 (blue)	BMPR1A Cancer vs. Normal (red)	BMPR1A (blue)	BMPR1B Cancer vs. Normal (red)	BMPR1B (blue)	BMPR2 Cancer vs. Normal (red)	BMPR2 (blue)
Bladder Cancer						2		4			1			2				
Brain and CNS Cancer	6						3				3	1	1	1	2		1	
Breast Cancer		7				2		5		3	1				4			
Cervical Cancer						1												
Colorectal Cancer		12		7	5			14		8	5							
Esophageal Cancer	3				2							4						
Gastric Cancer								1		2						1		
Head and Neck Cancer	5					1					1	1						
Kidney Cancer	3	1						3	1		1	2				2		
Leukemia	2	1		2								1					4	
Liver Cancer	1	1								1								
Lung Cancer		10						6		1	3							4
Lymphoma									1				2				2	
Melanoma		2				1						1						
Myeloma									2								1	
Other Cancer	3				1	1						1					2	1
Ovarian Cancer		1						1		2	2			1				
Pancreatic Cancer					2	1										1	1	
Prostate Cancer								1										
Sarcoma		5				2		1	1	5		1						
Significant Unique Analyses	23	40		9	10	11	3	36	4	22	17	12	3	4	6	4	11	5
Total Unique Analyses	430		375		444		436		447		460		418		414		446	

Analysis Type by Cancer	ACVR1 Cancer vs. Normal (red)	ACVR1 (blue)	ACVR1B Cancer vs. Normal (red)	ACVR1B (blue)	ACVR1C Cancer vs. Normal (red)	ACVR1C (blue)	ACVR2A Cancer vs. Normal (red)	ACVR2A (blue)	ACVR2B Cancer vs. Normal (red)	ACVR2B (blue)	ACVRL1 Cancer vs. Normal (red)	ACVRL1 (blue)	TGFBR1 Cancer vs. Normal (red)	TGFBR1 (blue)	TGFBR2 Cancer vs. Normal (red)	TGFBR2 (blue)	TGFBR3 Cancer vs. Normal (red)	TGFBR3 (blue)
Bladder Cancer			1					2								1		3
Brain and CNS Cancer				2		5		3	1	1			1				1	
Breast Cancer						6		1			1	2		1		9		16
Cervical Cancer	1			1									1	1				3
Colorectal Cancer				1		8		4	2			13	4				2	2
Esophageal Cancer	2														3		1	1
Gastric Cancer													1					
Head and Neck Cancer	1							1	1			1	2					5
Kidney Cancer				4														5
Leukemia		1	1	4			1		5			2		6		3	3	4
Liver Cancer										1								1
Lung Cancer				1	1				1			11				5		12
Lymphoma	5		3	3		1	5				8			3				1
Melanoma																		1
Myeloma	1						2		1									
Other Cancer	3		2	1					2		2		4					3
Ovarian Cancer		1		1				1	1			1				1		5
Pancreatic Cancer	1												1					
Prostate Cancer								1										1
Sarcoma	1			4												3		1
Significant Unique Analyses	15	2	7	19	1	20	8	13	14	2	11	30	14	11	3	22	7	64
Total Unique Analyses	459		445		239		438		453		440		432		396		462	
