# Supplementary figures and images for: Abnormal Expression and Prognostic Significance of Bone Morphogenetic Proteins and Their Receptors in Lung Adenocarcinoma
Source: Biomed Res Int. 2021 May 7;2021:6663990. doi: 10.1155/2021/6663990 (PMC8123996; doi:10.1155/2021/6663990)

A

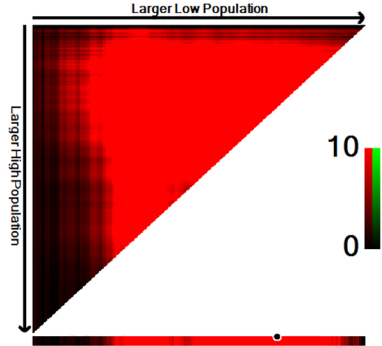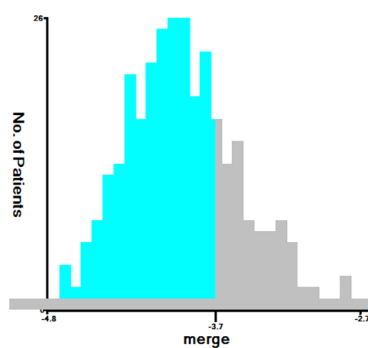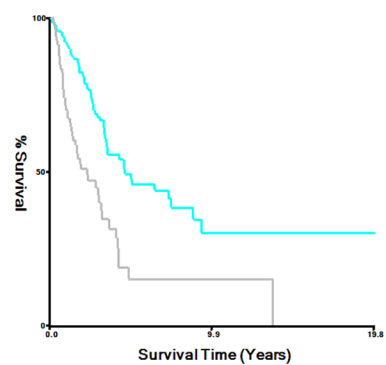

B

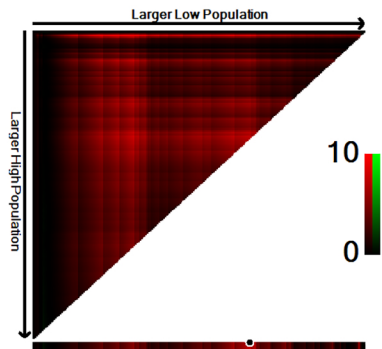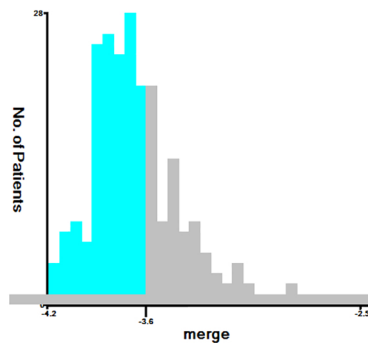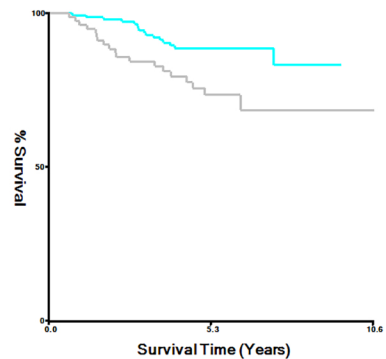

Supplement: Supplementary Materials — Figure S1: significantly changed BMPs/BMP receptors expression in different types of cancers. The color was determined by the highest gene rank percentile genes based on fold-change; red illustrated up-regulation and blue indicated down-regulation. BMP: bone morphogenetic protein. Figure S2: X-tile was used to determine the best cutoff value of risk scores in TCGA (a) and GEO (b). The cut-point was shown on a histogram of the entire cohort (middle panels). A Kaplan-Meier plot (right panels; the low subset = gray, the high subset = cyan). Figure S3: the risk scores of the three genes. From top to bottom, the distribution of risk scores, the survival status of each patient, and the heat map of mRNA expression levels of the three genes were displayed for TCGA (a) and GEO (b) database. [file 6663990.f1.zip › FigureS2 (2).pdf]

A TCGA

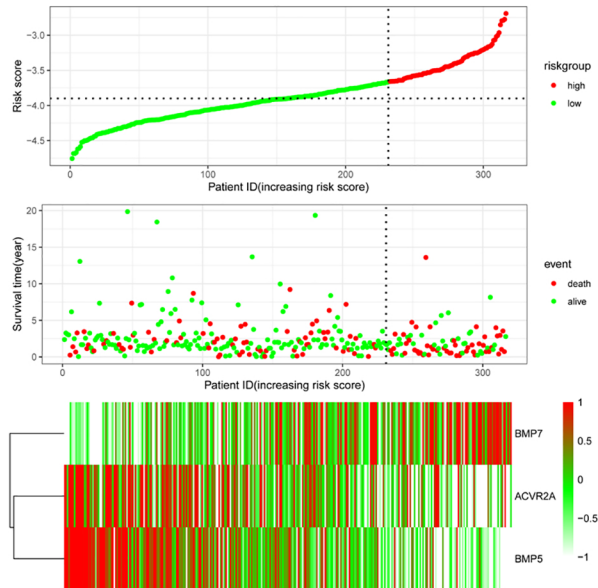

B GSE31210

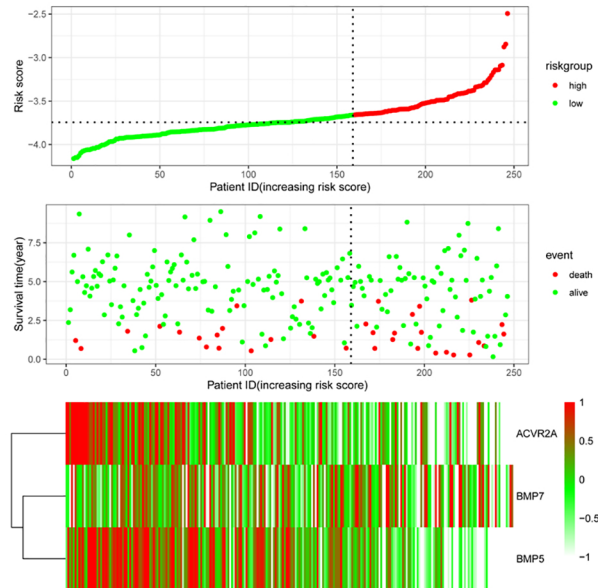

Supplement: Supplementary Materials — Figure S1: significantly changed BMPs/BMP receptors expression in different types of cancers. The color was determined by the highest gene rank percentile genes based on fold-change; red illustrated up-regulation and blue indicated down-regulation. BMP: bone morphogenetic protein. Figure S2: X-tile was used to determine the best cutoff value of risk scores in TCGA (a) and GEO (b). The cut-point was shown on a histogram of the entire cohort (middle panels). A Kaplan-Meier plot (right panels; the low subset = gray, the high subset = cyan). Figure S3: the risk scores of the three genes. From top to bottom, the distribution of risk scores, the survival status of each patient, and the heat map of mRNA expression levels of the three genes were displayed for TCGA (a) and GEO (b) database. [file 6663990.f1.zip › FigureS3 (2).pdf]
